# Severe Shivering as an Adverse Effect of Regadenoson Myocardial Perfusion Imaging

**DOI:** 10.7759/cureus.14091

**Published:** 2021-03-24

**Authors:** Tanjeev Ahmad, Juan Linares, Damian N Valencia, Ajay Agarwal

**Affiliations:** 1 Department of Internal Medicine, Division of Cardiovascular Medicine, Boonshoft School of Medicine, Wright State University, Dayton, USA; 2 Department of Internal Medicine, Division of Cardiovascular Medicine, Kettering Medical Center, Dayton, USA; 3 Department of Cardiology, Dayton Veterans Affairs Medical Center, Dayton, USA; 4 Department of Internal Medicine, Division of Interventional Cardiology, Boonshoft School of Medicine, Wright State University, Dayton, USA

**Keywords:** regadenoson, drug-related side effects and adverse reactions, myocardial perfusion imaging, shivering, mpi, cardiac stress testing

## Abstract

Regadenoson myocardial perfusion imaging (MPI) is a widely used screening study for patients with an intermediate pretest probability of coronary artery disease (CAD). Via selective agonism of the adenosine A2A receptor, regadenoson can induce coronary steal, revealing stenotic vessel territory through transient ischemia. Common side effects of this medication include chest pain, shortness of breath, nausea, vomiting, atrioventricular block, seizure, and allergic reactions. Here we present a case of severe shivering and chest tightness after the administration of regadenoson, along with a physiologic explanation and treatment.

## Introduction

Myocardial perfusion imaging (MPI) is a widely used diagnostic tool for patients with intermediate pretest probability for coronary artery disease (CAD). Considering the COVID-19 pandemic, MPI has gained popularity as exercise stress testing becomes more difficult due to mandatory mask use while running on a treadmill. [1] Regadenoson is an FDA-approved pharmaceutical agent commonly used during MPI. Although relatively safe, this drug has known cardiovascular and non-cardiovascular side effects. Of the cardiovascular effects, chest pain and shortness of breath are frequently documented, which can be explained by selective adenosine receptor (A2A) agonism and coronary steal. [2-3] Less frequently, episodes of myocardial ischemia, atrioventricular block, and myocardial infarction have been reported. [4] Rare non-cardiovascular side effects include gastrointestinal (nausea, diarrhea, and abdominal discomfort) and neurological (seizures and convulsions) symptoms. [3] Here we present a case of severe shivering and chest tightness after the administration of regadenoson, along with a physiologic explanation and proposed treatment.

## Case presentation

This patient is a 72-year-old male with a past medical history of obstructive sleep apnea, hypertension, hyperlipidemia, type 2 diabetes mellitus, and CAD status post percutaneous intervention with a drug-eluting stent to the left anterior descending (LAD) artery two years prior. He was referred for cardiac nuclear stress imaging for atypical symptoms (sub-sternal chest pressure present at rest and with activity, not relieved by rest). A review of systems was negative for any signs of infectious illness, and he denied any history of cold intolerance. Vital signs (126/78 mmHg, 77 beats/minute, 97% SpO2, 37° C) and physical examination before MPI were within normal limits. The room temperature was adequate (72 F). The patient was wearing a regular hospital gown and was covered with two sets of linen for comfort. He did not express feeling cold, and his skin appeared pink with no cyanosis or paleness. The MPI was performed with regadenoson 0.4 mg as per institutional protocol. Shortly after administration, the patient developed chest tightness and severe uncontrollable shivering. The shivering was generalized and vigorous, graded as 3 out of 4 by the shivering scale described by Guffin et al. [5] Severity of shivering on this scale is as follows; 0 = no shivering, 1 = occasional mild tremors in the jaw and neck, 2 = intensive tremors in the chest, 3 = intermittent vigorous generalized tremor, and 4 = continuous violent muscle activity. There was no change in the patient's body temperature. As per protocol, three 50 mg doses of aminophylline were given without any clinical improvement. A 25 mg intravenous (IV) injection of diphenhydramine was also given without relief of symptoms. After approximately 30 minutes, the patient’s chest pain and shivering gradually resolved. The patient was discharged home in stable condition. Follow-up elective cardiac catheterization revealed a patent previously placed stent in the LAD without any new obstructive coronary disease.

## Discussion

Regadenoson primarily has selective adenosine A2A receptor activity which predominantly affects the coronary artery smooth muscle cells. It also has some weak activity at the adenosine A1A, A2B, and A3A receptors. [6] A2A receptor agonism results in vasodilation of coronary vessels by activation of Gα and cAMP stimulation via adenylate cyclase production. [7] The A1A, A2A, A2B, and A3A receptors are broadly expressed in peripheral tissues of the cardiovascular, respiratory, renal, and immune systems. [8] In regards to the case presented, hypothermia could have occurred as a “protective” response to perceived severe noxious stimuli. Shivering may follow as an attempt to correct for the acute drop in body temperature. Being a weak agonist of A1A and A3A receptors, regadenoson can inhibit cAMP production in peripheral vessels, thereby restricting blood flow to the extremities, decreasing body temperature, and prompting the physiological shivering response. This effect has been well documented in animal models following the stimulation of various adenosine receptors. This hypothermic response does not occur in genetically modified mice lacking the involved receptors. [9] Aminophylline could have potentially reversed A1A and A3A agonist activity in our patient, permitting vasodilation and cessation of shivering with the restoration of normal body temperatures. The other medication given was diphenhydramine which could have also played a role in improving symptoms by reversing any activity at the histamine receptors. Diphenhydramine is an antagonist of the histamine-1 receptor which, if previously activated via an allergic reaction to regadenoson, could have led to this patient’s severe shivering. The FDA fact sheet on regadenoson describes tremor as a rare side effect that has been reported during the post-marketing phase. [10]

## Conclusions

Shivering is a rare side effect of regadenoson, thought to be a result of A1A and A3A adenosine receptor agonism, peripheral vasoconstriction, and the physiologic response to decreased body temperature. Our patient was given aminophylline and diphenhydramine which resolved symptoms after approximately 30 minutes. As regadenoson MPI becomes more frequent, the incidence of such side effects may also increase. It is essential that clinicians are aware of the potential side effects and reversal agents.
